# Driver Vision Based Perception-Response Time Prediction and Assistance Model on Mountain Highway Curve

**DOI:** 10.3390/ijerph14010031

**Published:** 2016-12-30

**Authors:** Yi Li, Yuren Chen

**Affiliations:** The Key Laboratory of Road and Traffic Engineering, Ministry of Education, College of Transportation Engineering, Tongji University, Shanghai 201804, China; chenyr@tongji.edu.cn

**Keywords:** perception-response time, driver vision, mountain highway curve

## Abstract

To make driving assistance system more humanized, this study focused on the prediction and assistance of drivers’ perception-response time on mountain highway curves. Field tests were conducted to collect real-time driving data and driver vision information. A driver-vision lane model quantified curve elements in drivers’ vision. A multinomial log-linear model was established to predict perception-response time with traffic/road environment information, driver-vision lane model, and mechanical status (last second). A corresponding assistance model showed a positive impact on drivers’ perception-response times on mountain highway curves. Model results revealed that the driver-vision lane model and visual elements did have important influence on drivers’ perception-response time. Compared with roadside passive road safety infrastructure, proper visual geometry design, timely visual guidance, and visual information integrality of a curve are significant factors for drivers’ perception-response time.

## 1. Introduction

A variety of studies are under way today in developing driver assistance systems. A driver assistance system aims to help drivers to drive more safely under various traffic environments. Modern technologies, such as video detection, Light Detection and Ranging (LiDAR), and V2X communication, have been tested and used in some cases [1]. However, a new question arises with such rapid development of information-based systems: how do intelligent driving systems (including assistance driving systems) deal with these forms of information? Will they respond like a machine or a human being? Responding like a machine means using a pre-set unchangeable process mode as the core of the system without consideration of drivers’ personal needs. A humanized system learns to respond in human recognition mode that takes both a driver’s perspective and real-time mechanical data into consideration, which would thereby provide suitable assistance according to different driving conditions. 

In this research study, an intelligent driving system is regarded as the interaction between a driver and a vehicle. The advanced driver assistance system (ADAS) has been improved a lot in recent years, with many features such as obstacle warning, collision warning, lane control and traffic light assistance strategy, to keep drivers informed of the latest information or warnings during the driving process [2,3,4,5]. In such systems, the so-called “Perception-response time” (PR time) or “Perception-response distance” is usually defined as an empirical value (e.g., 3 s) according to the Chinese Highway Standard [6] or a fixed range (e.g., 1.5 to 3.5 km) [7]. However, perception-response (PR) time as defined by Olson and Sivak is “the time from the first sighting of an obstacle until the driver applies the brakes” [8]. It was tested that the PR time of over 95% of tested drivers, including young and old, was 1.6 s. This indicates that the value suggested by the current national standard is improper, due to the loss of real individual data. However, timely, relevant, and comprehensive information is quite vital when providing drivers with more choices and perception-response time to an emergency situation [9].

As PR time varies among individuals, personal driving behavior should to be included in order to meet drivers’ humanized demands. Past studies mainly treat this factor as driving experience, which appeared to be a safe-relevant element in comparative study. It showed a positive impact in drivers’ response time to static roadway hazards [10]. By estimating the performance of novice, experienced, highly-experienced, and taxi drivers through video and eye movement data, Borowsky and Oron-Gilad revealed that drivers who had more driving experience were more skilled in the awareness of materialized hidden hazards [11]. Moreover, skilled drivers alter their driving behavior more frequently than the unexperienced ones to adapt accordingly to changing driving situations [12]. Although more driving experience would help drivers to respond more properly than new drivers [13], its impact differs among drivers. It is not always important in all situations. According to Sagberg and Bjørnskau [14], driving experience did not show a strong relationship with hazard perception time. It also did not show a significant influence on obvious risk perception. Some detailed methods have been introduced to describe the effects of different driving behaviors, such as driving habit graph (DHG) which can be utilized to explain different driving performances [15]. Naturalistic driving factors are also considered in individual-targeted driving response time studies, including safety margin adjusting [16], braking control [17], and eye-off-road ratio [18]. Drivers’ gaze data were also used in ADAS to identify simulation information for drivers and to make predictions of behaviors [19,20].

The factors above reflect drivers’ performance and differentiation, but ultimately the performance of drivers is still largely based upon the perception and decisions of drivers. A driving assistance system based on these elements loses the “Perception-Response” process. It cannot really “think” as a real driver from the first identification of a changing driving circumstance. Human-simulated ADAS needs to respond like a human in advance, such as the graphical model proposed by Oliver and Pentland who established the relationship between driver and vehicle with vehicle data, road geometric elements, and drivers’ vision data [21]. 

This research, as a further study, focuses on mountain highway curves in China where most accidents happened in 2014 among three kinds of highways curves: plain (3875 accidents), hill (3981 accidents), and mountain (6038 accidents) [22]. Different from the definition of perception-response time (PRT) in Olson and Sivack’ theory, the PRT in this study focused on “left/right curve” rather than “obstacle”. The PRT in their theory is an important component of stopping sight distance on a vertical curve (e.g., cresting a hill) to avoid collision. However, in this research we predict drivers’ response time from the curve appearance until the first reaction, during which they do not need to stop, but to drive safely. For the different driving purpose, we do not apply their theory in our PRT definition or prediction model which is only serves as a hint or piece of evidence for us to improve Chinese current national standard. The inner relationship between road information obtained by a driver’s vision and the perception-response time on a mountain highway curve is discussed. Based on on-board video, Catmull-Rom spline describes the driver-vision lane and corresponding elements are extracted. These visual factors and mechanical factors are then used in a multinomial log-linear model via neural network, which has also been verified with a series of field tests. This work serves as the basis of human-like ADAS that takes actual perception-response time into consideration.

**Figure 1:**
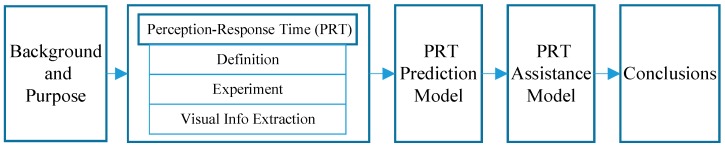
Workflow of the research.

This paper consists of five sections as shown in Figure 1.

The first section introduces the background and purpose of this study. The second section illustrates the definition of PR time in this research. The experiments data and visual information are also explained. The third section builds a multinomial log-linear prediction model for PR time. A corresponding assistance model is also established and tested in the fourth part. The last section concludes the key points of this study.

## 2. Experiments and Data

### 2.1. Definition of Perception-Response Time on Mountain Highway Curves

Traditional perception-response time on a highway according to the Chinese Highway Standard is divided into three parts: driver’s sensing time, reaction time, and braking time. The first two parts show the human factors in perception-response time. A mechanical process is considered in the third part. It is always considered as a value (3 s) in the design and evaluation of a highway. However, highways with various combinations of characteristics/or different environmental conditions can result in different driving behaviors. For instance, drivers may take a variety of strategies (e.g., steering, lifting the foot from accelerator, or braking) when a highway curve appears, which will lead to different perception-response times.

**Figure 2:**
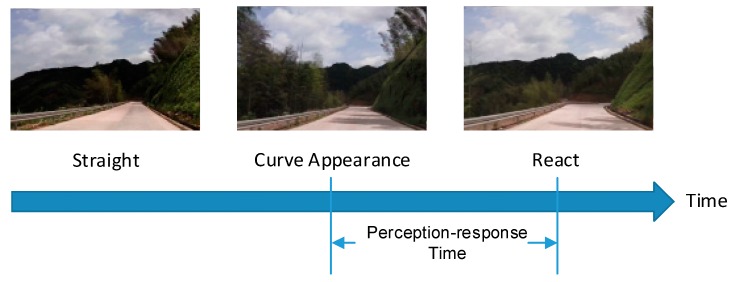
Illustration of perception-response time on a mountain highway curve.

In this study, the perception-response time on a mountain highway curve is defined as the duration from the first sight of a curve’s appearance in a driver’s sight to the moment of the driver’s reaction (braking or turning). Figure 2 shows the meaning of this term. This identification is different from the perception concept in Embodied Cognition Theory [23], which reflects the overall response period from the curve appearance to the first detection moment of driver reaction (either steering or braking).

### 2.2. Experiments

To understand the impact of different perception-response (PR) times on driving behaviors on mountain highway curves, 220 field experiments were conducted during the daytime on mountain highway curves (design speed: 40 km/h) in Lishui and Suichang city, Zhejiang Province, China. Based on field conditions, tested curves were divided into six types. Detailed parameters are listed in Table 1.

A total of 32 drivers who had over three years of driving experience (Mean: 11.4, S.D.: 2.4) took part in the experiments. They were between the age of 28 and 40 (Mean: 38.8, S.D.: 3.5). During the tests, they were not assigned with any task. Each driver faced the same route and were asked to drive on the six types of curves respectively. A driving recorder (GARMIN GDR35) was fixed on windshield to record the front view. During the tests, a built-in sensor that was synchronized with the camera and fixed on the steering wheel recorded the vehicle’s mechanical changes. Table 2 shows the detailed data collected by sensors.

**Figure 3:**
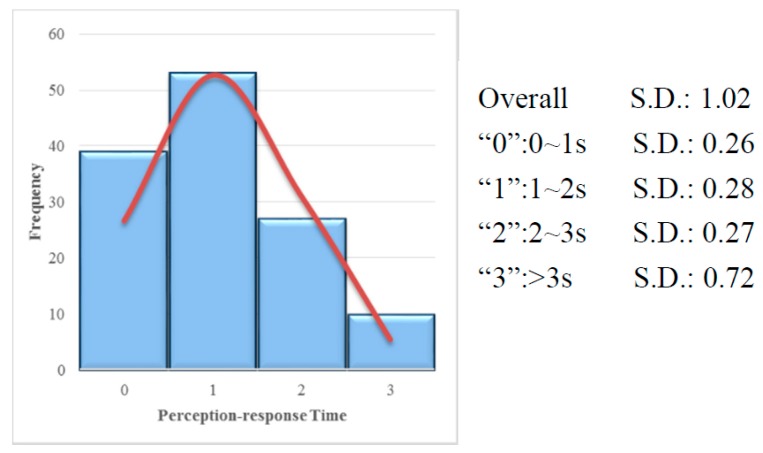
Distribution of perception-response time (all tests).

According to the video and vehicle data, the perception-response time of each test can be obtained. After eliminating incomplete and wrong records, 129 tests were applied in the following study. The population distribution of perception-response time shown in Figure 3 indicates that most tested drivers were inclined to respond between 1 and 2 s.

**Figure 4:**
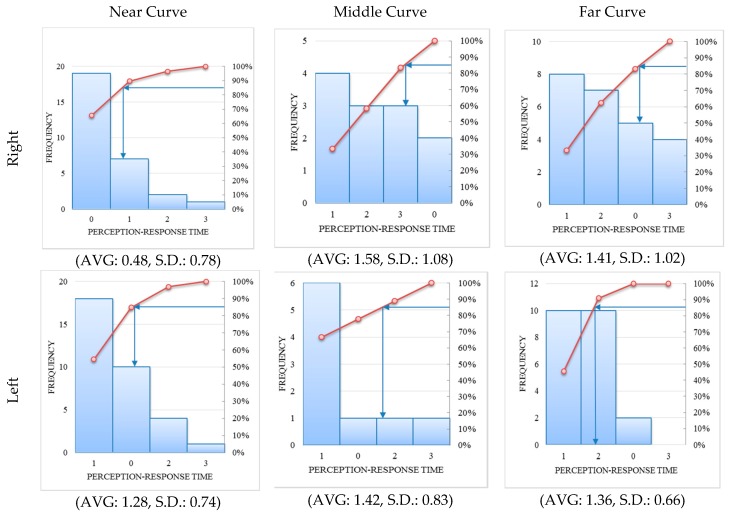
Histogram and cumulative frequency curve of perception-response time on six types of curves.

However, different scenarios would have diverse impacts on drivers’ perception-response times. A comparative analysis was done to reveal drivers’ perception-response times on the six kinds of curves. Figure 4 shows the histogram and cumulative frequency curve [24,25,26] of the tested curves.

The average and standard deviation of the PR times show that drivers reacted quickly on near curves, especially on near right curves. The variation of PR times on near right curves is also quite low. This means most drivers’ perception-response times on such curves are relatively consistent. However, the middle curves, including right and left curves, tended to result in longer PR times than other types of curves, which was not expected. It can also be seen from Figure 4 that 85% of drivers reacted within two seconds when faced with a near curve on a mountain highway. On middle-right curves, over 30% of participants’ PR times were among 1~2 s and the 85th quantile of PR times was over 3 s. The test results also show that drivers’ PR times were usually under 3 s (0~3 s) on middle-left curves and far-right curves. On far-left curves, they tended to react within 1~3 s. Three mechanical coefficients of variation on six types of curves during the turning process are listed in Table 3.

CV = σ/μ(1)

where σ is standard deviation; μ is average value.

Three kinds of coefficients were relatively low among 85% tested drivers, which is in accordance with the distribution of PR time. This means that a suitable PR time would result in good turning process (low CV). In the following assistance model, the 85th quantile of PR time will be regarded as the boundary value for the recommended range.

### 2.3. Visual Information Extraction

**Figure 5:**
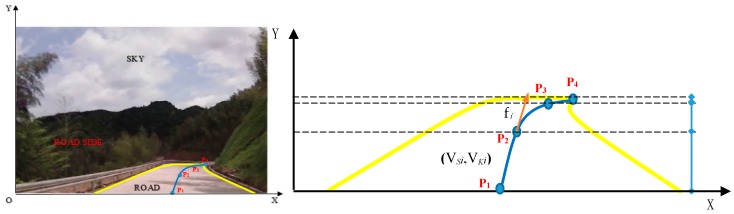
Driver-vision lane model based on driver’s visual perception (unit: pixel).

According to our previous study (see Yu et al. [27]), the road alignment in a driver’s vision can be described as a driver-vision lane (the blue line in Figure 5) during the process of driving. The driver-vision lane model is based on Catmull-Rom spline to fit with a driver’s visual perception (a detailed explanation of this model is available in Yu et al. [27]). Perception characteristics of road alignment in this research include the following elements obtained by the driver-vision lane model. The bottom-left corner of the on-board driving recorder video is set as the origin of coordinate. 

There are four control points (P*_i_*, *i* = 1, 2, 3, 4) on Catmull-Rom spline. The value of ① accumulated spline length (S*_i_*, *i* = 1, 2, 3); ② spline length between P*_i_* and P*_i_*_−1_ ($V_{S_{i}}$, *i* = 1, 2, 3); ③ tangent direction angle of P*_i_* (f*_i_*, *i* = 1, 2, 3, 4); ④ curvature rate between P*_i_* and P*_i_*_−1_ ($V_{K_{i}}$, *i* = 1, 2, 3) are decided by P*_i_*. The relationship between these elements is as follows.

(2) $$V_{S_{i}}=S_{i}-S_{i-1}\;V_{K_{i}}=\frac{f_{i}-f_{i-1}}{V_{S_{i}}}$$

where $V_{S_{i}}$ is the visual curve length between control point P*_i_* and P*_i_*_+1_ (pixels); $V_{K_{i}}$ is the visual curve curvature between control point P*_i_* and P*_i_*_+1_.

There are two rules to judge a curve based on an image with the elements above:

(1) Curve appearance: On a straight highway, the tangent direction angles are normally equal to each other. When there is a curve ahead, there will be a change in f_4_.
(2) Right/left curve: f_4_ > 0 and $V_{K_{3}}$ < 0 means right curve; f_4_ < 0 and $V_{K_{3}}$ > 0 means left curve.

Apart from detecting the elements of the driver-vision lane model, we also classified the visual information of traffic/road environment into six major parts. Each part consists of four sub-classifications (see in Table 4).

## 3. Perception-Response Time Prediction Model

In this study, a driver’s perception-respond (PR) time is predicted by three parts: traffic/road environment information, driver-vision lane model, and mechanical status (last second). Traffic/road environment is a uniform factor for each driver. It provides drivers with similar driving tasks. However, similar or even the same curve conditions did not result in the same PR times according to the tests. This means there are some other factors effecting drivers’ PR processes, such as the driver-vision lane model. It is the impact of lane alignment on drivers, and last second speed and acceleration adjustment is another facet considered in the prediction model. 

**Figure 6:**
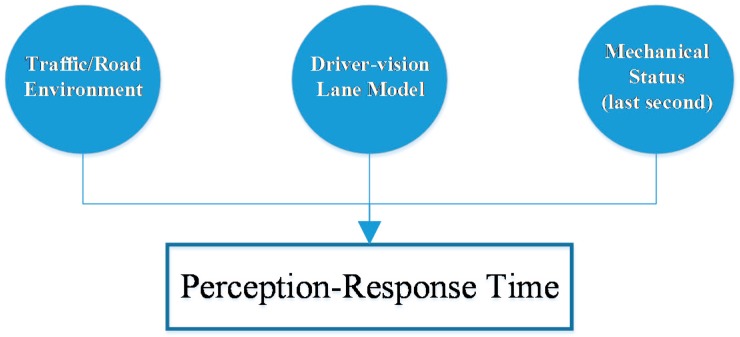
Three parts in perception-response time prediction model.

In Figure 6, the three parts all have a certain impact on drivers’ PR times, for drivers’ responses depend on some knowledge: what are the environmental conditions (traffic/road environment), what is the shape of the next curve (driver-vision lane model), and what is the status of the vehicle (mechanical status). Detailed parameters of these three parts are listed in Table 5.

A multinomial log-linear model via a neural network can be established to quantify the weights of the parameters above. After 790 iterations, the converged model results are shown in Table 6.

The model provided here is a multinomial log-linear regression model based on one-hidden-layer neural network. The *p*-value is an index of the multinomial log-linear model, which is a post-calculated value. AIC is Akaike Information Criterion, which is used as a measure of the relative quality of statistical models for a given set of data. In this model, $V_{K_{2}}$ and $V_{K_{3}}$ of driver-vision lane have the most significant impact on drivers’ PR times, especially on the PR times during 2~3 s and >3 s. Compared with $V_{K_{2}}$, $V_{K_{3}}$ shows a greater determining effect on PR time. Although it shows a negative impact on the third category, one unit change (Odds Ratio [28]) of it would result in the most significant change on PR time compared with other factors. Hence $V_{K_{3}}$ is a determining factor. A large $V_{K_{3}}$ would lead to a sharp drop in drivers’ PR times. Highway access is the third important factor which would result in lower PR times (<3 s). Among the protective roadside marking and infrastructure, warning pier is the most effective way to guide drivers. The existence of corrugated-steel guardrails has a negative impact on PR times over 1 s, so that they would accelerate the PR process. Concrete guardrails and traffic markings/signs have similar low influence on PR times. Each additional opposite vehicle results in additive negative effects. This means that a high volume of opposite traffic increases driving task difficulty and decreases PR times. All the distance of curve appearance parameters are positive, and they have larger impacts on longer PR time. This conforms to common knowledge. However, curve distance between 30 m–50 m has a greater positive influence on both short and long PR time compared with curve distance over 50 m. This reveals that drivers responded dispersedly in such circumstance. PR times increase with large longitudinal impact force (steep slope) and decrease with vertical impact force (rough road surface). This shows the impact of road geometry on drivers’ PR times. Drivers’ real-time operation consists of speed and acceleration changing rate, but they are not significant in the prediction model.

## 4. Perception-Response Time Assistance Model

### 4.1. Model Structure

**Figure 7:**
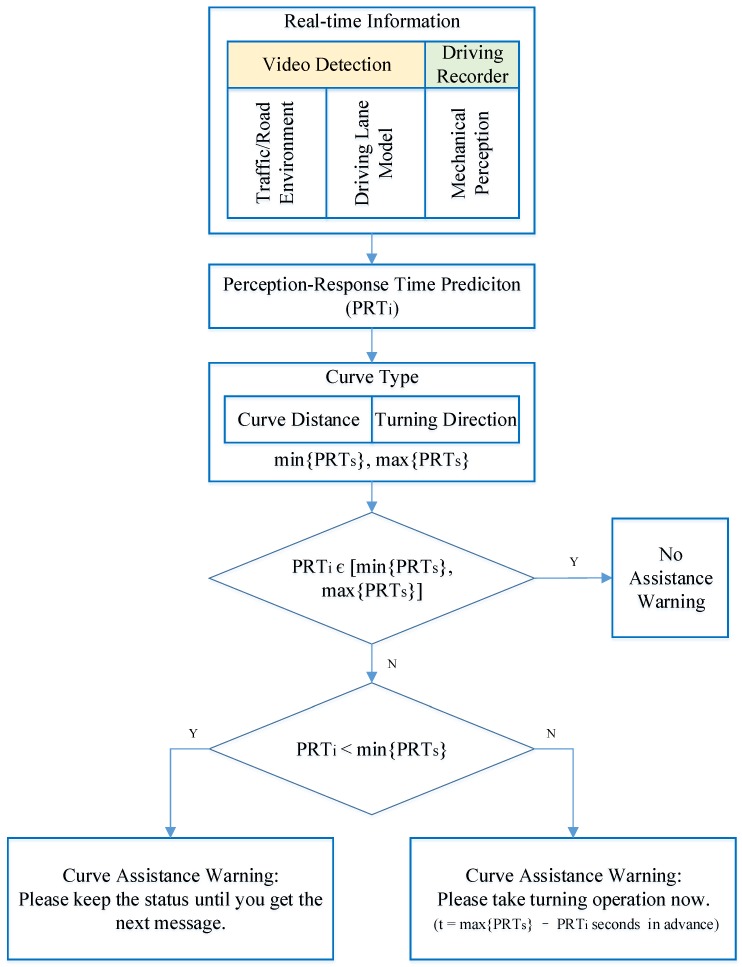
Perception-response time assistance model flowchart.

According to the PR time prediction model and the recommended range of PR times introduced in Section 2.2, this section will build a PR time assistance model to help drivers to respond timely and properly on a mountain highway curve. Figure 7 describes the flowchart of the assistance model.

### 4.2. Model Calibration

**Figure 8:**
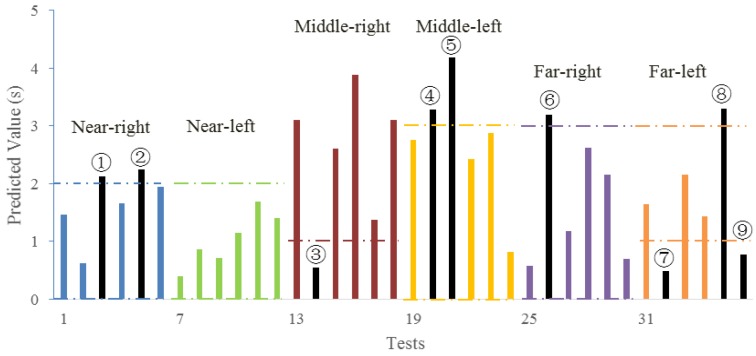
Results of 6 × 6 test. The numbered bars represent the tests with inappropriate PR time.

There were six tests conducted on each type of curve. Six drivers did one test on six types of curves respectively. The PR time prediction results are shown in Figure 8.

In Figure 8, the black bar stands for the tests of which the predicted PR time is inappropriate (beyond or below the recommended range). The dash line is the recommended range of PR time according to the 85th quantile in Figure 4. The PR times of most drivers were within this range. On near-left curves, no sample was beyond the recommended range. All cases (black bars) with unsuitable PR time were detected by the assistance model (see Table 7).

Among the five curve types, the far-left curves tended to result in a large deviation on PR times, followed by the middle-right curve. This means that when a far-left curve appeared, some drivers would take reaction within 1 s and other drivers tended to respond far more slowly. Average dispersion change of CV had more improvement on right-turning curves than left-turning curves after assistance, especially on near-right curves. This would result from the fact that the right-turning process has more flexibility than left-turning process according to the traffic rule in China. The possibility of an unsafe turning process (large value of three CVs) would increase with different PR times. Hence proper assistance strategy can help drivers to behave well in such circumstances.

## 5. Conclusions 

This study defined the concept of perception-response time (PR time) on mountain highway curves. Field tests were conducted with a driving recorder to record drivers’ real-time driving data both before and during the turning process. As drivers’ visual information is an important factor in perception-response time prediction in this study, driver-vision lane model was used to extract the elements of visual information from the sight of the drivers. To predict the perception-response times, a multinomial log-linear prediction model with the elements of traffic/road environment, driver-vision lane model, and mechanical status was presented. A corresponding assistance model was also illustrated to help drivers to have more proper perception-response times on mountain highway curves.

The results showed that the same road situation could result in various understandings regarding the interpretation of perception-response times in this work. Compared with the length of driver-vision lane, curvature rate (especially $V_{K_{3}}$) had more impact on PR times. This indicates that drivers’ PR process is mostly based on the visual information integrality of curves. Uncertainty of the curve shape and curvature rate will lead to uncertain PR times, such as observed with the middle curve (middle-VAR: 1.162, near-VAR: 0.609, far-VAR: 0.826). Meanwhile, the effect of road safety infrastructure are less significant than that of driver-vision lane elements. Therefore, good road visual geometry is more effective than passive road safety infrastructure in driving behavior guidance.

On the other hand, this study verified the need for establishing a personal-targeted assistance model, which took drivers’ personal behavior and visual information into consideration. This is more humanized than a traffic-rule-based system. Although the model in this study has been proven valid, the absolute data and the meaning obtained should be considered only as relative result [29] due to the limitation of low-frequency data. More high-frequency and accurate biological information detectors would contribute to future studies. The change of eye gaze position/area would also help to illustrate drivers’ different perception processes to establish a more personalized assistance system. In the future, complex driving behavior and driver-based decision processes will be analyzed and quantified.

## Figures and Tables

**Table 1:** Categories of Tested Curves.

Curve Category	Distance	Curve Direction	Curve Category	Distance	Curve Direction
1	Near (<30 m)	Right	4	Middle (30~50 m)	Left
2	Near (<30 m)	Left	5	Far (>50 m)	Right
3	Middle (30–50 m)	Right	6	Far (>50 m)	Left

Note: “Distance” in the table means how far the curve beginning point is away from the test car when it comes into view.

**Table 2:** Data Collected by Inner Sensor of Driving Recorder.

Parameter	Accuracy	Frequency
Longitude and Latitude	-	1 hz
Speed	1 km/h	1 hz
Vertical Acceleration	0.001 g	1 hz
Lateral Acceleration	0.001 g	1 hz
Longitudinal Acceleration	0.001 g	1 hz

Note: “g” means gravity (9.8 m/s^2^).

**Table 3:** Average Value of Three Mechanical Coefficients of Variation (CV) on Six Types of Curves.

CV	PR Time	Type of Curves											
		(1)		(2)		(3)		(4)		(5)		(6)	
		V.	N.	V.	N.	V.	N.	V.	N.	V.	N.	V.	N.
Lateral force	0	1.78	19	0.43	18	3.93	4	0.53	6	2.24	8	0.32	10
	1	0.89	7	0.4	10	2.59	3	0.58	1	1.99	7	0.22	10
	2	1.67	2	0.24	4	2.85	3	0.49	1	3.23	5	0.44	2
	3	4.15	1	0.81	1	0.69	2	0.17	1	2.22	4	NO RECORDS	
Lateral deviation	0	0.01	19	0.17	18	0.04	4	0.01	6	0.05	8	0.02	10
	1	0.02	7	0.02	10	0.02	3	0.02	1	0.06	7	0.03	10
	2	0.02	2	0.09	4	0.03	3	0.05	1	0.05	5	0.03	2
	3	0.05	1	0.5	1	0.08	2	0.01	1	0.03	4	NO RECORDS	
Speed	0	2.36	19	0.72	18	0.88	4	0.64	6	1.09	8	2.48	10
	1	1.15	7	0.81	10	0.59	3	0.43	1	1.22	7	1.34	10
	2	3.24	2	1.92	4	0.38	3	0.24	1	1.22	5	1.17	2
	3	2.34	1	3.14	1	2.33	2	1.02	1	1.68	4	NO RECORDS	

Note: (1) six types of scenarios are mentioned in Figure 4; (2) grey area covers 85% participants; (3) V. means “Value”; (4) N. means “Number of drivers”.

**Table 4:** Visual Information Classification of Traffic/Road Environment.

No.	Classification	Sub-Classification			
		0	1	2	3
1	Access	None	Far	Near	Village nearby
2	Sign/Marking	None	Lane marking	Information sign	Danger warning sign
3	Lane width	<3.5 m	3.5–3.75 m	>3.75 m	Multi-lane
4	Passive road safety infrastructure	None	Warning pier	Corrugated-steel guardrail	Concrete guardrail
5	Vision shelter	None	Tree/House	Mountain/Tunnel	Vehicle ahead
6	Road surface	Smooth	Mottled	Bumpy	Sand gravel

**Table 5:** Explanation of Parameters Considered in the Prediction Model.

Category	Explanation	Parameters	Variable Type
Traffic/road environment information	The area proportion of front view	Sky proportion	Continuous
		Road proportion	
	Pavement type	Sand-gravel surface	Dummy: 1
		Bituminous pavement	0
	Passive road safety infrastructure	Warning pier	Dummy: 100
		Corrugated-steel guardrail	010
		Concrete guardrail	001
		No road safety infrastructure	000
	Lane marking and yield sign	Traffic marking and sign	Dummy: 1
		No marking or sign	0
	Whether access before curve	Access exists	Dummy: 1
		No access	0
	Opposite traffic	Number of opposite vehicles	Continuous
	Curve distance	Distance (>50 m)	Dummy: 10
		Distance (30–50 m)	01
		Distance (<30 m)	00
Driver-vision lane model	Visual curve length	S_1_ (m)	Continuous
		S_2_ (m)	
		S_3_ (m)	
	Visual curve curvature	|$V_{K_{1}}$|	Continuous
		|$V_{K_{2}}$|	
		|$V_{K_{3}}$|	
Mechanical status	Impact force of last second	Impact force (vertical)	Continuous
		Impact force (longitudinal) Impact force (latitudinal)	
	Speed of last second	Speed (km/h)	Continuous
	Acceleration change of last second	Acceleration changing rate (m/s^3^)	Continuous

**Table 6:** Perception-Response Time Prediction Model on Mountain Highway Curves.

Data Source	Parameters		Perception-Response Time					
			1 ^a^		2 ^a^		3 ^a^	
			Coefficient	*p*-Value ^b^	Coefficient	*p*-Value	Coefficient	*p*-Value
	(Intercept)		45.500	0.000 ***	31.164	0.000 ***	82.597	0.000 ***
Video detection	Sky proportion		4.015	0.257	−1.902	0.661	48.616	0.005 **
	Road proportion		−8.468	0.252	−6.321	0.522	−92.144	0.000 ***
	Sand-gravel surface		−0.193	0.817	−0.830	0.448	21.907	0.000 ***
	Warning pier		−29.157	0.000 ***	−27.469	0.000 ***	26.006	0.000 ***
	Corrugated-steel guardrail		−3.021	0.042 *	−1.087	0.455	−4.157	0.000 ***
	Concrete guardrail		−2.328	0.018 *	−2.717	0.020 *	10.485	0.066 ^#^
	Traffic marking and sign		−2.682	0.044 *	−2.441	0.134	8.080	0.313
	No access		−34.343	0.000 ***	−34.401	0.000 ***	80.602	0.000 ***
	Number of opposite vehicles		−0.885	0.345	−1.257	0.294	−49.488	0.000 ***
	Distance (>50 m) ^c^		1.470	0.051 ^#^	2.640	0.003 **	15.777	0.001 ***
	Distance (30–50 m)		2.266	0.026 *	2.526	0.057 ^#^	24.819	0.000 ***
	S_1_ (m)		−0.312	0.241	0.203	0.506	−3.710	0.000 ***
	S_2_ (m)		0.026	0.931	−0.354	0.309	2.642	0.002 **
	S_3_ (m)		0.062	0.735	0.109	0.590	−0.089	0.917
	|$V_{K_{1}}$|		2.885	0.854	25.855	0.020 *	60.162	0.000 ***
	|$V_{K_{2}}$|		−14.996	0.000 ***	169.928	0.000 ***	451.889	0.000 ***
	|$V_{K_{3}}$|		76.444	0.000 ***	121.068	0.000 ***	−875.740	0.000 ***
Driving recorder	Last second	Impact force (vertical)	−2.719	0.543	2.340	0.668	−88.139	0.000 ***
		Impact force (longitudinal)	5.364	0.235	−1.338	0.821	34.066	0.001 ***
		Speed (km/h)	−0.045	0.271	0.064	0.196	−1.399	0.000 ***
		Acceleration changing rate (m/s^3^)	0.203	0.597	0.162	0.709	1.972	0.384
	AIC (Akaike Information Criterion)		325.897					
	Adjusted R^2^		0.78					
	Precision		72.0%		83.3%		98.9%	

Note: ^a^ Perception-response time categories: 1 (1~2 s), 2 (2~3 s), 3 (>3 s); 0 (0~1 s) is the control group; ^b^ *p*-Value significance: “***”: 0 ≤ *p* ≤ 0.001, “**”: 0.001 < *p* ≤ 0.01, “*”: 0.01 < *p* ≤ 0.05, “^#^”: 0.05 < *p* ≤ 0.1;; ^c^ “Distance” in the table means how far the curve is when it comes into view. It was classified into: >50 m, 30–50 m, <30 m, considered as dummy variables.

**Table 7:** Perception-Response Time Assistance Model Performance.

Curve Type	No.	PR Time Deviation	Warning Success (0-Fail, 1-Success)	Average Dispersion Change of CV ^a^ after ASSISTANCE
Near-right	①	0.13 s	1	↓19%
	②	0.25 s	1	↓24%
Middle-right	③	−0.46 s	1	↓8%
Middle-left	④	0.28 s	1	↓10%
	⑤	1.18 s	1	↓11%
Far-right	⑥	0.2 s	1	↓9%
Far-left	⑦	−0.51 s	1	↓6%
	⑧	0.3 s	1	↓12%
	⑨	−0.23 s	1	↓3%

Note: ^a^. Mechanical coefficients of variation are described in Section 2.2.
